# What Is the Relevance of the Tip-Apex Distance as a Predictor of Lag Screw Cut-Out?

**DOI:** 10.1371/journal.pone.0071195

**Published:** 2013-08-28

**Authors:** Jérôme M. Goffin, Paul J. Jenkins, Rishikesan Ramaesh, Pankaj Pankaj, A. Hamish Simpson

**Affiliations:** 1 Department of Orthopaedic Surgery, The University of Edinburgh, Edinburgh, United Kingdom; 2 Orthopaedics and Trauma, The Royal Hospital for Sick Children, Edinburgh, United Kingdom; 3 Medical School, The University of Edinburgh, Edinburgh, United Kingdom; 4 School of Engineering, The University of Edinburgh, Edinburgh, United Kingdom; 5 Department of Orthopaedic Surgery, The University of Edinburgh, Edinburgh, United Kingdom; Faculté de médecine de Nantes, France

## Abstract

Using a simple mathematical formulation, the relationship between the position of the lag screw tip (relevant to both intramedullary and extramedullary devices) and the concept of tip-apex distance (TAD) was derived. TAD is widely used in operating theaters as a surgical guideline in relation to the fixation of trochanteric fractures, and in clinical studies as a predictor of lag screw cut-out. In order to visualize better this concept, the locus of points having the same TAD was plotted and the dependence of TAD on the location of the lag screw tip was also reported. It was shown that TAD should be adjusted for the size of the femoral head (a variable which varies a lot according to the sex of the patient) while no correlation was found between TAD and bone morphometry indices obtained from micro-CT data (BV/TV and Tb.Th). Therefore, these results seem to suggest that TAD lacks mechanical justification and that predictors which are based on mechanical properties, such as bone density, should be investigated further.

## Introduction

Cut-out of the sliding hip screw from the femoral head accounts for up to 84% of the failures of fixation of extracapsular proximal femoral fractures [1]. Intramedullary nailing also suffers from the same complication since Lobo-Escolar et al. reported a cut-out rate of 3.6% in a study including 916 hip fracture patients [2].

The tip-apex distance (TAD), suggested by Baumgaertner et al. [3], is currently considered to be the gold standard amongst predictors of lag screw cut-out. This predictor is relevant to the positioning of both intramedullary nails and sliding hip screws. Large-scale clinical studies attempted to determine the best cut-off value for TAD under which the risk of cut-out is minimized [4]. TAD is defined as the sum of the distances between the tip of the lag screw and the apex of the femoral head, as measured on the anterior-posterior (AP) view (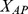) and on the lateral view (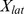), both distances being corrected for radiograph magnification by using the true diameter of the lag screw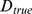 as a reference, i.e.
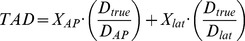
(1)


On the other hand, operative guidelines often recommend to avoid placing the lag screw in a superior position and to prefer instead a middle or even inferior position within the femoral head [4]. Some clinical studies presented their results by dividing the femoral head into nine zones (anterior to posterior and inferior to superior), according to Cleveland et al. [5], in order to visualize the position of the lag screw [3], [4].

A mathematical formulation to calculate TAD knowing the coordinates of the tip of the lag screw and geometrical parameters of the proximal femur (such as anteversion angle, neck-shaft angle and head diameter) can be a useful research tool whenever the AP and lateral views, which are typically used to calculate TAD, are not available. If 3D data is the only information available, for instance coming from a CT scan, CAD software or a finite element (FE) model, this formulation can model the rotations of the C-arm in order to recover numerically the components of TAD in the missing AP and lateral views. Such a tool could also help to measure TAD more accurately in clinical trials (from a 3D scan of the hip), since TAD was shown to be heavily dependent on hip rotation [6], and therefore associated with potentially large errors when calculated from AP and lateral views.

The aim of this study is to derive the relationship between TAD and lag screw position using a simple mathematical formulation and correlate this with micro-CT data obtained from osteoporotic femoral heads. Micro-CT is indeed a powerful imaging tool which can shed some light on the micro-architecture of trabecular bone [7]–[10]. It is hypothesized that, even though it is a widely used predictor of cut-out, TAD does not correlate with morphometry indices describing the micro-architecture of the femoral head.

## Materials and Methods

**Figure 1:**
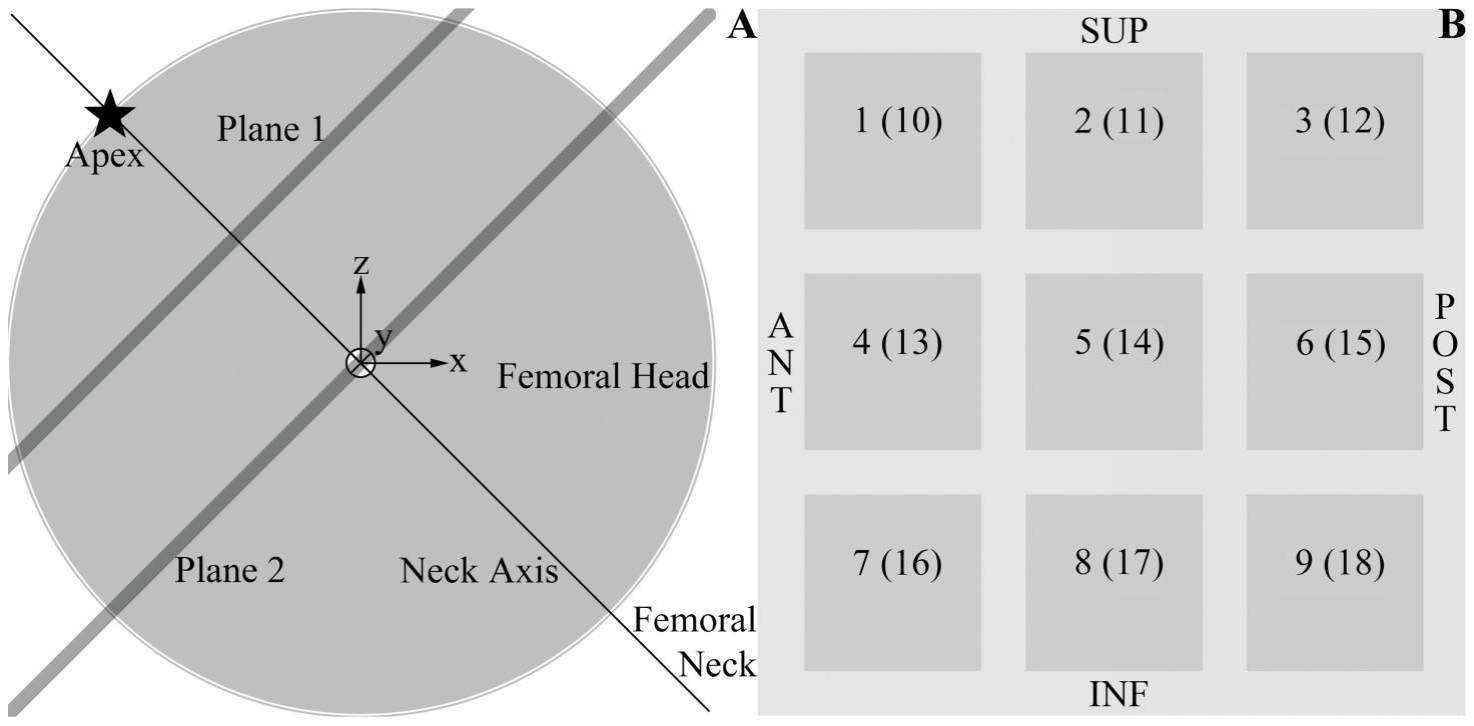
Spherical model of the femoral head. (A) The femoral head was modeled as a sphere centered at the origin of a right-handed Cartesian coordinate system, and whose apex was depicted as a star in this projection of the head on an AP view. The femoral neck axis (at an angle of 135 degrees with the femoral shaft) was drawn as a thin black line going through the apex and the center of the sphere, while the planes of interest for the results shown in Figure 3 were represented as thick lines. (B) Cross-section through the femoral head showing the labeling of the different regions of the femoral head (ROIs) at two levels, planes 1 and 2 as shown in (A). ROIs 1–9 belong to plane 1 and ROIs 10–18 to plane 2.

**Figure 2:**
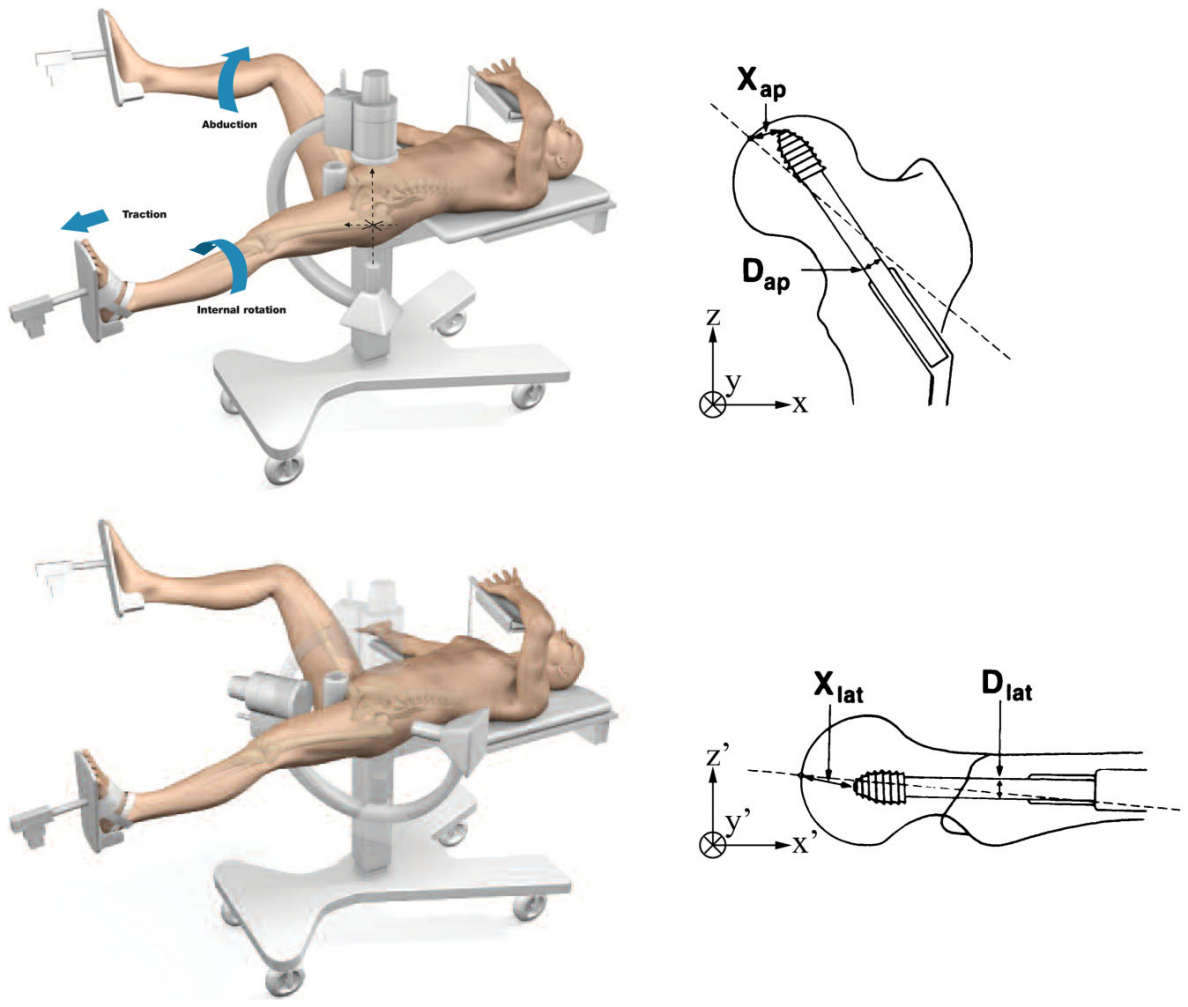
C-arm for AP and lateral views. The position of the C-arm was matched to a typical view of the proximal femur with an implanted hip screw, both AP and lateral. The corresponding components of the tip-apex distance were also shown to illustrate Eq. 1. Finally, the two coordinate systems *xyz* and *x′y′z′* were also described. The axes should actually have their origin at the center of the femoral head, but were translated to make the diagrams less cluttered. *xyz* are related to an AP view while *x′y′z′* result from two rotations applied to *xyz* (see Materials and Methods section). Reproduced with permission from Stryker and Baumgaertner [3].

The femoral head was considered to be a perfect sphere with a mean diameter of 47 mm [11] and centered at the origin of a right-handed three-dimensional Cartesian coordinate system. Axes *xyz* (Figures 1 and 2) correspond to an AP view where the anteversion angle has been cancelled out by internally rotating the hip. Hence, they are related to anatomical landmarks as follows: the *z*-axis corresponds to the direction of the femoral shaft, the *x*-axis points laterally in the plane of the femoral neck, and the *y*-axis is at right angle to the other two axes. For the purpose of showing the effect of femoral head diameter on TAD, the diameter was assumed to fall within the following clinical range: 35 to 59 mm [12]. The component of the tip-apex distance in the AP view was taken as the true distance between apex and lag screw tip projected on plane *xz* (i.e. plane of the femoral neck) while the TAD component in the lateral view was computed as the distance projected on the appropriate perpendicular plane (Supporting information S1). A neck-shaft angle of 135° was assumed, which is in line with the range of values observed anatomically [13]. Calculations and related plotting were performed using Matlab (The MathWorks, Natick, USA). The range and distribution of TAD was calculated for each of four different distances of the apex from the tip; these distances were chosen to represent a range from a quarter of the radius *r* of the femoral head to the whole radius of the femoral head (i.e. *r*/4, *r*/2, 3*r*/4 and *r*). For a femoral head of 47 mm in diameter, this equates to radial distances of the tip to the apex of 5.875, 11.75, 17.625 and 23.5 mm.

Micro-CT scans (Skyscan 1172, Skyscan Ltd, Belgium) were obtained with a 100 kV beam at a resolution of 30 µm for 18 cubic regions of interest (ROI) of 5 mm^3^ in volume from the femoral head of 6 patients who had undergone hemiarthroplasty or total hip replacement. The heads were stored in formaldehyde prior to scanning. The location of these ROIs can be described as follows: let us consider two planes perpendicular to the axis of the femoral neck (the projection of these planes on the AP view is depicted as thick grey lines on Figure 1), plane 2 going through the center of the femoral head (assumed to be spherical) and plane 1 going through the midpoint between the center and the apex of the head. In each of these planes, 9 ROIs are distributed in a 3×3 arrangement according to the Cleveland femoral head dividing system [5]. Plane 1 contains ROIs 1–9 and plane 2, ROIs 10–18 (Figure 1). Imaging data were then converted to morphometric indices describing the structure of cancellous bone using CTAn v1.1 (Skyskan Ltd,). Correlation between TAD and these morphometric indices was tested using Pearson's correlation coefficient and by assuming that a *p*-value less than 0.05 was enough to show that the correlation found was statistically significant. This methodology has previously been developed and reported by our group [14].

## Results

**Figure 3:**
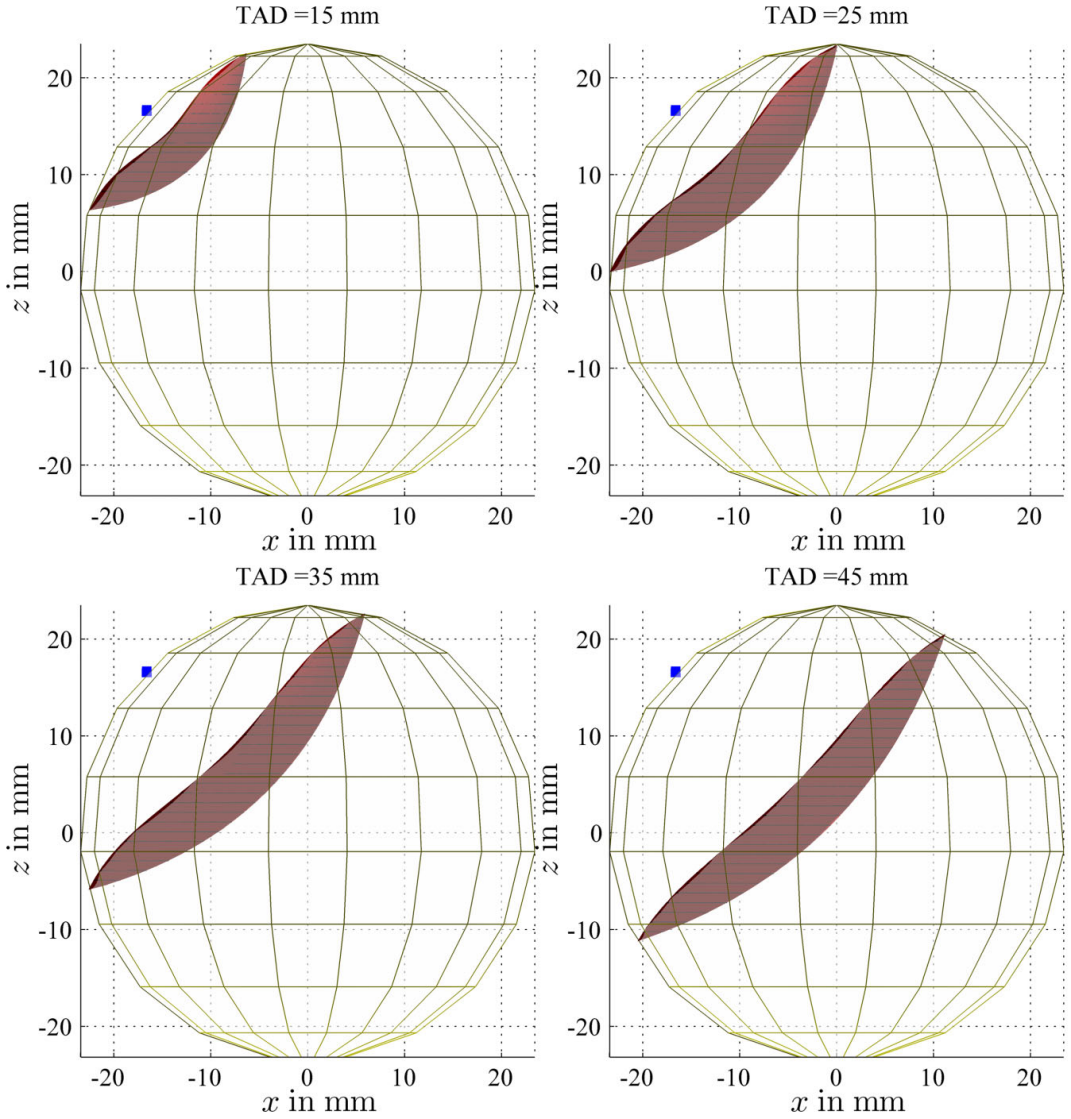
AP view of the locus of points with a constant TAD. The locus of points (projected on an AP view) with a TAD value equal to 15, 25, 35 and 45 mm was drawn as a red surface for a spherical femoral head with a diameter of 47 mm. The blue dot depicts the position of the apex of the head. Contour lines with a constant *z* value were also plotted to visualize better the shape of the surface.

Many different lag screw positions can lead to the same TAD, as illustrated in Figure 3, which shows the locus of points in the femoral head (diameter of 47 mm) having the same TAD, with the apex represented as a blue dot. The locus was drawn for the recommended TAD cut-off value of 25 mm [15] and for other values spanning the whole range of clinically relevant TAD, as published by Lobo-Escolar et al. [2], i.e. 15 mm, 35 mm and 45 mm. In this large clinical study, the TAD value was 23.8±8.5 mm for the control group and 32.2±11.4 mm for the cut-out group.

**Figure 4:**
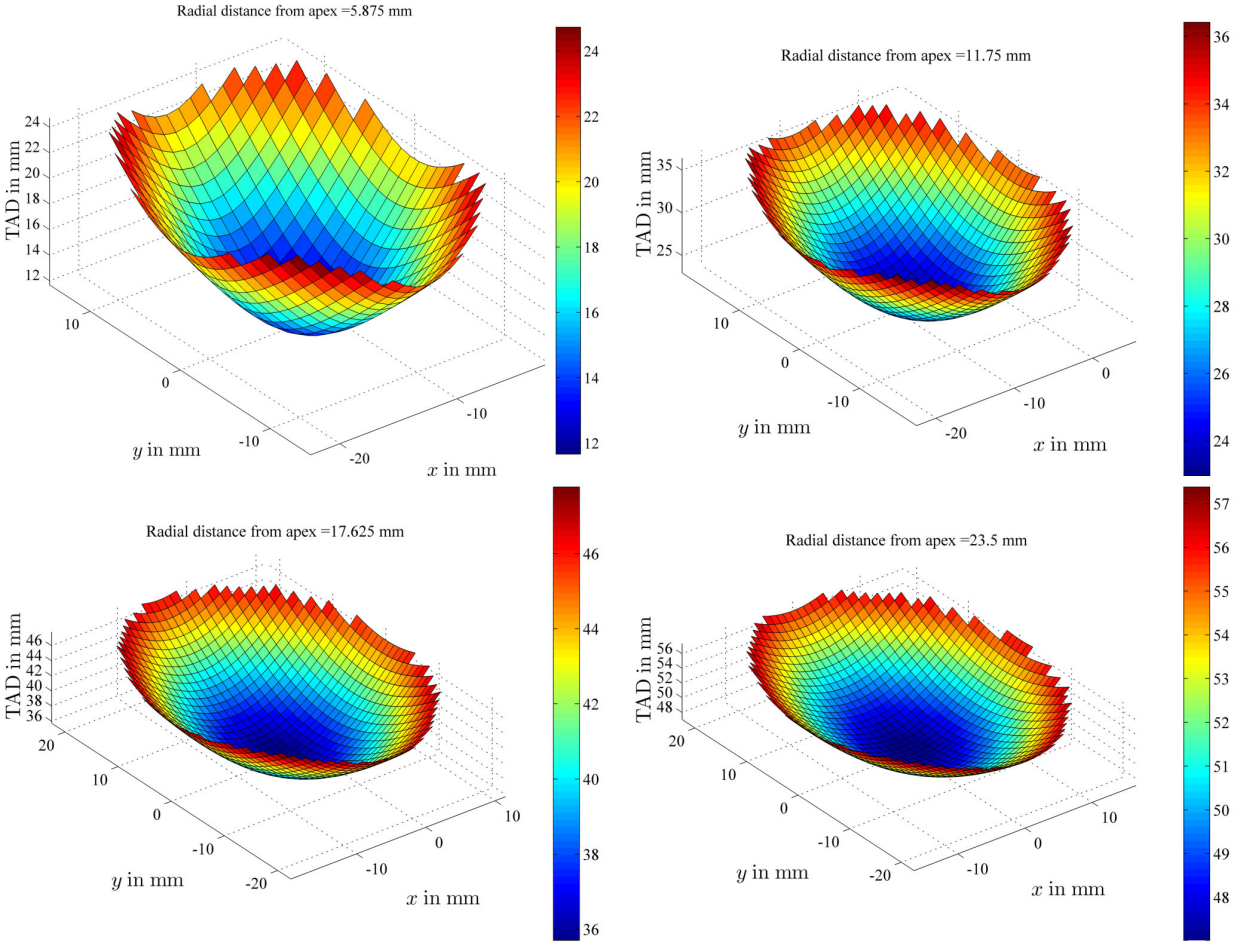
TAD values within a given plane. TAD values were plotted for all the possible positions of the lag screw tip within planes whose projection on plane *xz* was characterized by an angle of 45 degrees with the *x*-axis. These planes were further defined by the radial distance which separates them from the apex of a femoral head of 47 mm in diameter. The color bars provide a visual representation of the TAD values in mm.

The relationship between TAD and lag screw position was then reported (Figure 4) for a femoral head with a diameter of 47 mm under the following assumption: the lag screw tip was moved within planes 1 and 2, shown in Figure 1, and two additional planes, one between planes 1 and 2 and one between the apex and plane 1, i.e. four different planes parallel to each other, with a radial distance from the apex equal to *r*/4 (5.875 mm), *r*/2 (11.75 mm), 3*r*/4 (17.625 mm) and *r* (23.5 mm), or in other words from a plane very close to the apex to one going through the center of the femoral head. At a radial distance of 5.875 mm, the TAD values range from 12 to 24 mm, at 11.75 mm, the TAD ranges from 24 to 36 mm, at 17.625 mm, the TAD ranges from 36 to 46 mm and at 23.5 mm, the TAD ranges from 48 to 57 mm. For the same radial distance from the apex, the TAD values can change by 100% if the radial distance amounts to 5.875 mm, by 50% at 11.75 mm, by 30% at 17.625 mm and by 20% at a radial distance of 23.5 mm (Figure 4).

**Figure 5:**
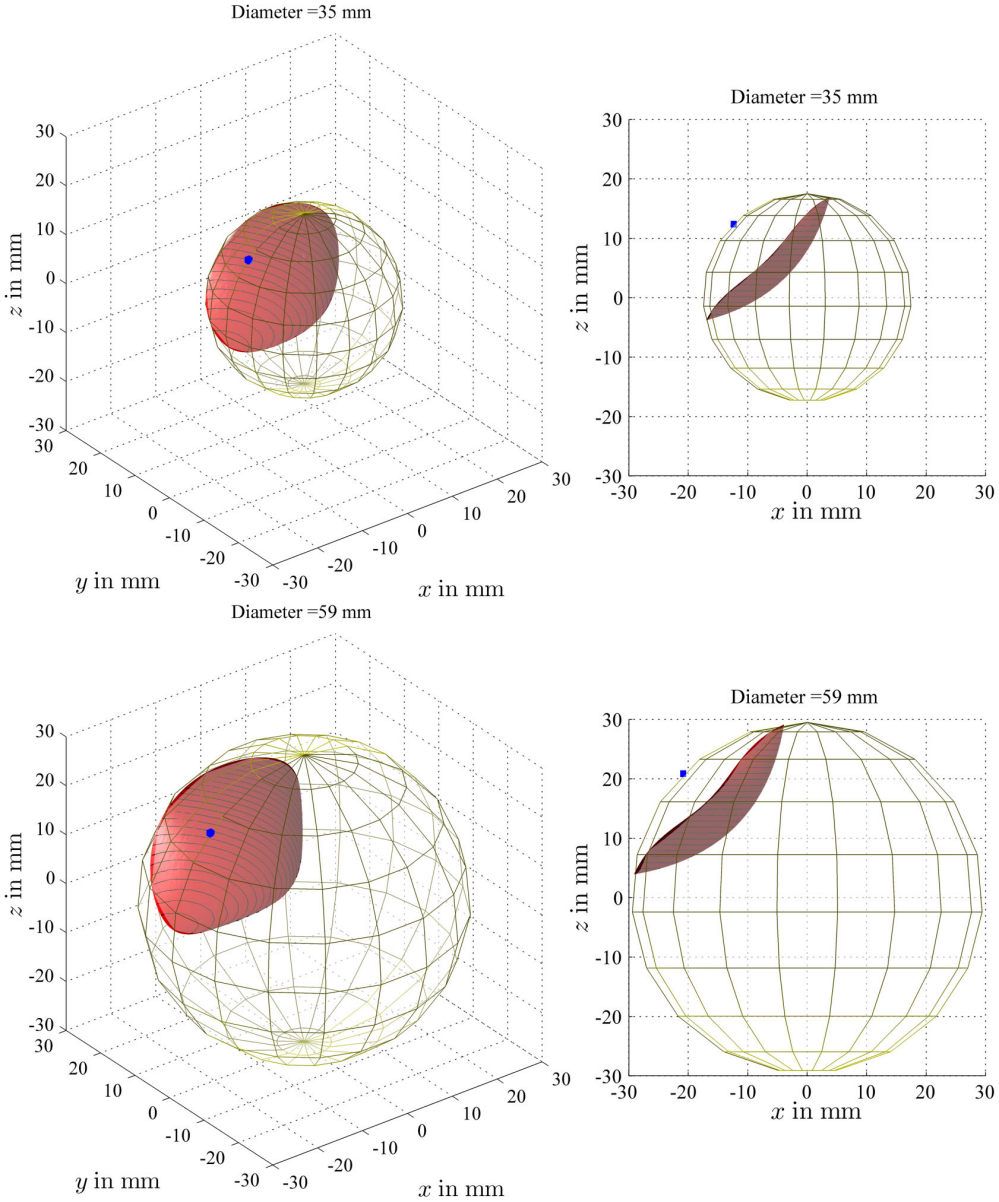
Locus of points with a constant TAD for different head diameters. The locus of points with a TAD equal to the recommended threshold value of 25 mm was plotted, both as a 3D view and in an AP view, for two extremes of the range of head diameters, i.e. 35 mm and 59 mm.

**Figure 6:**
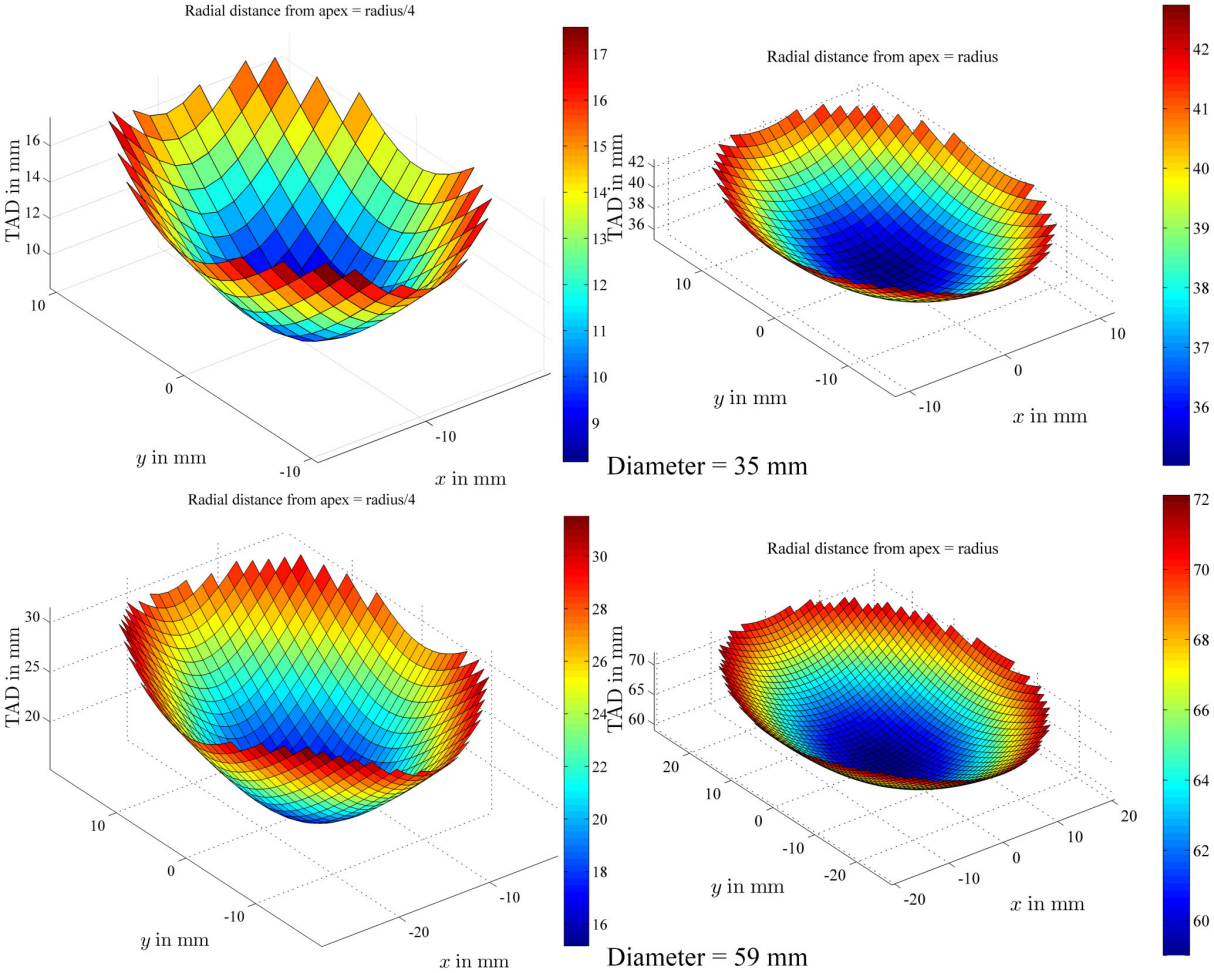
TAD values within a given plane for different head diameters. For two femoral heads with a diameter of 35 mm and 59 mm respectively, the TAD values were plotted for a lag screw tip located anywhere within a plane defined by a radial distance from the apex equal to *r*/4 and *r* (same planes as described for Figure 4).

The effect of head diameter on TAD was illustrated in Figure 5, where the locus of points with a TAD value of 25 mm was drawn for the two extremes of the range of head diameters (35 and 59 mm, according to an anatomical study on hundreds of specimens [12]). In Figure 6, the TAD values were calculated for a lag screw tip within two planes (plane 2 on Figure 1 and the parallel plane at a radial distance from the apex equal to *r*/4), as explained for Figure 4. For the plane close to the apex (*r*/4) in a small head, the TAD values range from 9 to 17 mm while for a large head, the TAD ranges from 16 to 30 mm. This showed that there is a large variation (70% of the radius of the femoral head for a small head of 35 mm to 40% for a large head of 59 mm) in relative radial distance of the apex to the tip for a given TAD and that it is highly dependent on femoral head diameter.

**Figure 7:**
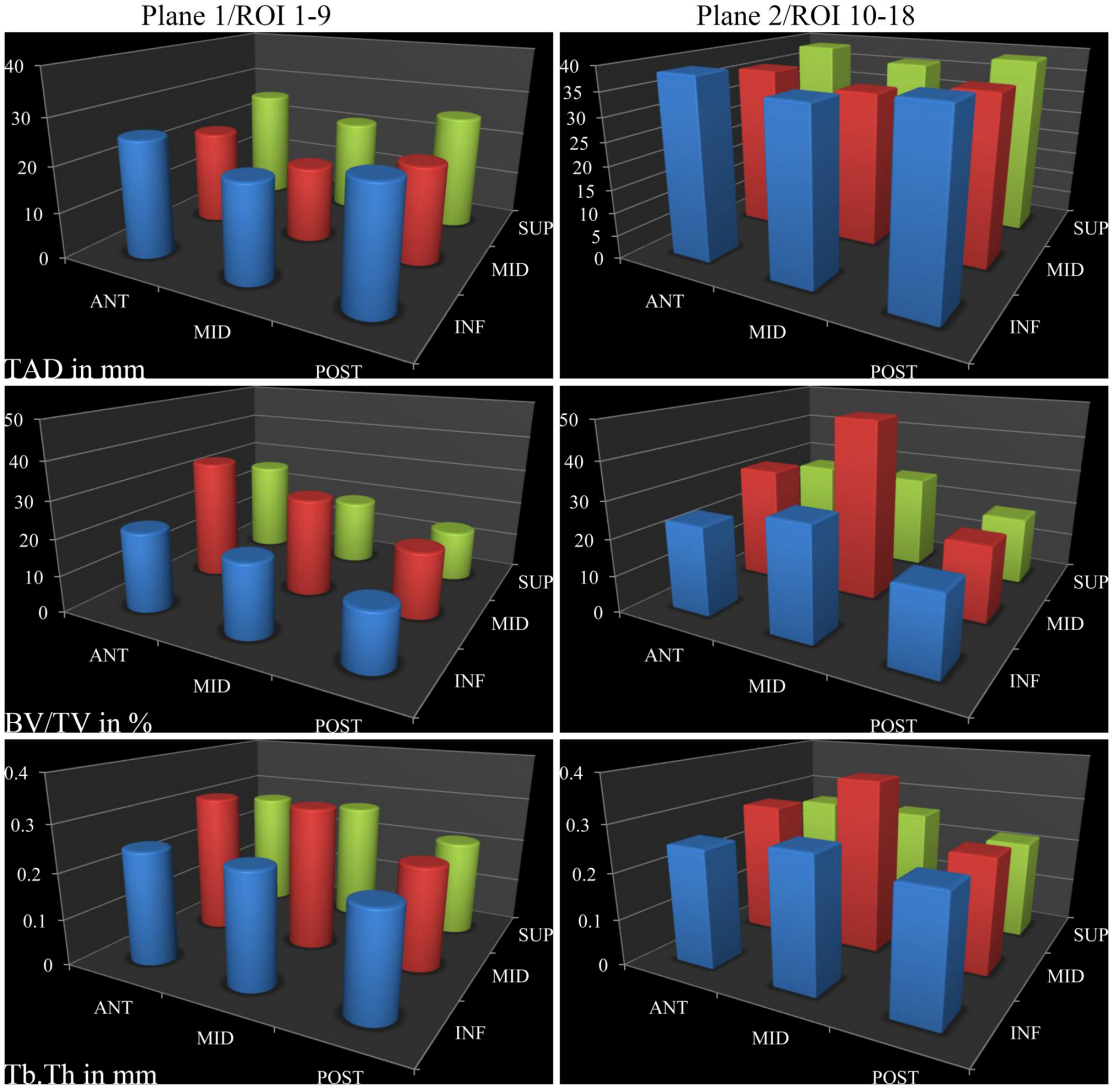
Relationship between micro-CT data and TAD. Tip-apex distance (TAD in mm), ratio of bone volume to total volume (BV/TV in %) as well as trabecular thickness (Tb.Th in mm) were represented for 18 cubic regions of interest (ROI) in planes 1 and 2, as depicted in Figure 1.

Finally, taking advantage of micro-CT scan data of femoral heads, the relationship between bone properties and TAD was assessed and reported in Figure 7. In particular, the following bone morphometry indices were considered: BV/TV (bone volume over total volume in percent) and Tb.Th (trabecular thickness in mm). There was no correlation between TAD and BV/TV (r = 0.17, p = 0.49) or TAD and Tb. Th. (r = −0.03, p = 0.89).

## Discussion

TAD had previously been found to be correlated to the amount of hip rotation [6]. In light of this result, the axis of the femoral neck was assumed to be parallel to the coronal plane, i.e. the hip was internally rotated.

The description of the whereabouts of the screw tip for a given TAD shows that having an extremely inferior position of the screw tip, as often recommended by clinical studies, can lead to very high TAD values (TAD of 45 mm on Figure 3). Inclusion of such equations in a surgical navigation system linked to the C-arm display could benefit the surgeon in the operating theater in order to target a given TAD value, provided this predictor remains the gold standard in the future.

Figure 4 demonstrates that, in a given plane across the head (at 45 degrees with the *x*-axis), TAD always reaches a minimum value for lag screw positions on the femoral neck axis, going through the apex and the center of the femoral head. TAD values considerably increase when the lag screw is moved away from that femoral neck axis, especially when the radial distance between the tip of the screw and the apex is small (upper left graph of Figure 4). Some clinical studies recommend positioning the lag screw in the inferior part of the femoral head, while keeping TAD below the accepted threshold. Figure 4 shows that this is almost a self-contradictory guideline, or at least very difficult to implement [16].

Tanner et al. reported the results of a clinical study running over a 5-year period, during which patients over the age of 50 were admitted for hip fractures in a gender ratio of 3∶1 (women to men) [17]. This supports the fact that hip fractures in elderly men are not an isolated problem and therefore, adjusting the concept of TAD to the size of the femoral head could be beneficial since femoral head diameter varies greatly according to sex [12]. Indeed, Figure 6 shows that TAD can almost double depending on the size of the head for equivalent lag screw positions (plane through the center of the head or plane defined by a radial distance of *r*/4 away from the apex).

Regarding the micro-CT results illustrated in Figure 7, BV/TV measures the fraction of mineralized bone found in a given volume of interest. It is a measure related to bone mineral density [9]. Tb.Th quantifies the thickness of trabeculae, and as such is a key measure for the description of the structure of cancellous bone [10]. Trabecular connectivity density [8] and the level of anisotropy [7] did not vary much across the head and were therefore not reported. Furthermore, Pearson's correlation coefficient ranges by definition from −1 to 1, i.e. from anti-correlation to correlation while no correlation at all would give a coefficient equal to zero. Thus, TAD was found not to be correlated with BV/TV (Pearson's correlation coefficient = 0.17 with a *p*-value of 0.49) or with Tb.Th (Pearson's correlation coefficient = −0.03 with a *p*-value of 0.89). However, lag screw cut-out is a purely mechanical issue. In fact, this complication occurs because the bone superior to the lag screw thread is too weak to sustain compressive strains exerted by the lag screw. Therefore, if TAD is not correlated with morphometry indices, which are themselves related to bone strength, the question arises as to whether TAD is indeed an appropriate predictor of cut-out. This lack of correlation suggests a rationale to start investigating predictors which are based on mechanical assumptions, such as bone density for instance. This could lead to a better prediction of cut-out and benefit those patients at risk by putting greater emphasis on the choice of implants and their positioning.

Hsueh et al. demonstrated the strength of TAD as a predictor of lag screw cut-out by reporting their results as follows: they claimed that for 17% of patients in the cut-out group, TAD was less than 25 mm whereas amongst 16% of patients for whom the fracture healed without cut-out, TAD was greater than 25 mm (up to 45 mm according to their histogram) [4]. They also reported that below 15 mm, no patient experienced cut-out. This result means that, even though the threshold of 25 mm is adequate to obtain two different groups (cut-out vs. no cut-out) with a statistically significant *p*-value, there is a large penumbra, i.e. a range of TAD values between 15 mm and 45 mm, where it is impossible to predict confidently the outcome for a specific patient. However, as previously mentioned, cut-out is a mechanical complication. Therefore, we believe that it should be possible to find a predictor of cut-out which conveys a clearer message for that range of TAD values.

To the best of our knowledge, there are not many studies which question the status of TAD as a reliable predictor of cut-out. A recent article by Herman et al. concluded that TAD values above 25 mm did not predict failure [18]. This study was, however, looking at a very specific intramedullary implant (PFN) with a lag screw and an antirotation screw, quite different from the more conventional implants (with a single screw in the head) usually included in studies on TAD. In particular, because of the presence of two screws in the head, the standard concept of tip-apex distance might not necessarily be adequate or at least more difficult to apply, i.e. TAD can only refer to a single screw while anchorage of the proximal femoral nail is likely influenced by the presence of both screws.

## Conclusion

This study was an attempt to provide better understanding of the relationship between a clinically important predictor of lag screw cut-out, i.e. the tip-apex distance, and the position of the lag screw tip. TAD is not an ideal concept, and as such has shortcomings. In particular, it does not take into account the size of the femoral head which can dramatically vary between male and female patients. Furthermore, no correlation was found between TAD and morphometry indices obtained from micro-CT data. Since cut-out is a purely mechanical problem, these results suggest that the concept of tip-apex distance, which does not seem to be related to any mechanical properties, might not be an ideal predictor of cut-out, especially if TAD falls in the penumbra zone between 15 and 45 mm. This provides a strong rationale for investigating predictors linked, for instance, to bone density.

## Supporting Information

Supporting Information S1 **Additional details regarding the rotations employed to model the movement of the C-arm were described in supporting information S1.**
(DOCX)
