# Supplementary material for: What Is the Relevance of the Tip-Apex Distance as a Predictor of Lag Screw Cut-Out?
Source: PLoS One. 2013 Aug 28;8(8):e71195. doi: 10.1371/journal.pone.0071195 (PMC3756032; doi:10.1371/journal.pone.0071195)
Supplement: Supporting Information S1 — Additional details regarding the rotations employed to model the movement of the C-arm were described in supporting information S1. (DOCX) [file pone.0071195.s001.docx]

The component of the tip-apex distance in the AP view was taken as the true distance between apex and lag screw tip projected on plane *xz* (i.e. plane of the femoral neck) while the TAD component in the lateral view was computed as the distance projected on plane *x’z’* after having applied two rotations to axes *xyz*. The transformation into axes *x’y’z’*, resulting from this set of rotations, models the rotation of the C-arm in the operating theater. A rotation of 45 degrees in plane *xz* around the *y*-axis was assumed to account for the fact that the lateral view is parallel to the femoral neck. This led to a coordinate system referred to as *x’yz’*. A second rotation of 90 degrees around *x’* in plane *yz’* was considered to model the rotation of the C-arm (Figure 2) and led to the final coordinate system (*x’y’z’*). This neck-shaft angle of 135 degrees, besides being within the range of values observed anatomically [[1](#_ENREF_1)], was specifically chosen to prove analytically that the numerical calculations were correct. In fact, with such rotations, a point located at the apex will have a TAD value equal to zero and a point located at the center of the femoral head will have a TAD equal to twice the radius of the head.

In 3D, to apply a rotation to a system of axes, rotation matrices can be used to transform the coordinates of any point of interest. For a rotation of angle $\theta$ around *x* (respectively *y*), the matrix has the following form

$R_{x}\left(\theta\right)=\left[\begin{matrix} 1 & 0 & 0 \\ 0 & \cos\theta& -\sin\theta\\ 0 & \sin\theta& \cos\theta\end{matrix}\right]$ $R_{y}\left(\theta\right)=\left[\begin{matrix} \cos\theta& 0 & \sin\theta\\ 0 & 1 & 0 \\ -\sin\theta& 0 & \cos\theta\end{matrix}\right]$.

**References:**

1. Wright D, Whyne C, Hardisty M, Kreder HJ, Lubovsky O (2011) Functional and anatomic orientation of the femoral head. Clin Orthop Relat Res 469: 2583-2589.
